# Description of total population hospital admissions for cleft lip and/or palate in Australia

**DOI:** 10.1186/s12903-015-0144-1

**Published:** 2015-12-07

**Authors:** Jonathan Y. J. Lo, Nicky Kilpatrick, Peter Jacoby, Linda M. Slack-Smith

**Affiliations:** School of Dentistry M512, The University of Western Australia, 35 Stirling Hwy, Crawley, WA 6009 Australia; Melbourne Dental School, University of Melbourne, Melbourne, Victoria Australia; Telethon Kids Institute, The University of Western Australia, Perth, Western Australia Australia

**Keywords:** Epidemiology, Australia, Congenital anomalies, Hospital admissions, Orofacial cleft, Total-population data

## Abstract

**Background:**

Orofacial clefts are a group of frequently observed congenital malformations often requiring multiple hospital admissions over the lifespan of affected individuals. The aim of this study was to describe the total-population hospital admissions with principal diagnosis of cleft lip and/or palate in Australia over a 10 year period.

**Methods:**

Data for admissions to hospitals were obtained from the Australian Institute of Health and Welfare National Hospital Morbidity Database (July 2000 to June 2010). The outcome variable was a hospital separation with the principal diagnosis of cleft palate, cleft lip or cleft lip and palate (ICD-10-AM diagnosis codes Q35-Q37 respectively). Trends in rates of admission and length of stay by age, gender and cleft type were investigated.

**Results:**

A total of 11, 618 admissions were identified; cleft palate (4,454; 0.22 per 10,000 people per year), cleft lip (2,251; 0.11) and cleft lip and palate (4,913; 0.25). Admission age ranged from birth to 79 years with males more frequently admitted. Most admissions occurred prior to adolescence in cleft palate and cleft lip and through to late teens in cleft lip and palate, declining for all groups after 25 years.

**Conclusions:**

This study identified population level trends in hospital separations for orofacial cleft diagnosis in Australia.

## Background

Clefting of the lip and/or palate is the most common congenital facial malformation worldwide. Available international data suggest that an orofacial cleft occurs in around one in 700 live births worldwide with birth rates varying from 3.13 (per 10,000 population) in South Africa to 19.05 births in Japan [1, 2]. The care of these infants would normally start prenatally and continue from birth through to adulthood and generally involves large multi-disciplinary teams of clinicians including surgeons, physicians, nurses, dentists and a range of allied health professionals [3]. Consequently, affected individuals typically engage with a wide range of health services from a very early age with repeated admission to hospital, primarily for surgical procedures to correct the structural defects, a necessary feature of their care pathway. The number, timing and nature of these surgeries varies around the world between cleft teams and is influenced by the type of cleft, the existence of co-morbidities and referral to, and availability of, services [4]. In addition to the elective and cleft related admissions, other interventions such as medical investigations, illness, pathology or accidental injury not necessarily directly related to the cleft anomaly may also result in hospital admission.

Little is known about the patterns of hospitalisation for those born with a cleft of the lip/palate [4] and yet a hospital admission is associated with significant costs, both financial and psychosocial to the individual, their family and the community. In the United States there is an 8–25 fold increase in medical care costs associated with being born with an orofacial cleft over the first ten years of life [5]. Such discrepancies may also not be limited to childhood; with a recent Danish study suggesting that these trends continue well in to adulthood, a fact which adds further support to the concept that orofacial clefting is associated with additional health risks across the entire life course [6]. The causal mechanism for orofacial cleft and associated co-morbidities remain unclear and there is a lack of comprehensive understanding of the patterns of health service utilisation by this population.

Historically, studies of congenital malformations such as orofacial clefting have been limited to hospital-based series. In the 1980s, birth defect registries were established in Australia, along with associated monitoring programs that overcame the limitations of previous descriptive studies [7, 8]. Since then, further improvements in the methods used and quality of total population data has made these datasets a valuable tool for answering a number of important research questions with data being increasingly available to clinicians [9]. The Australian Institute of Health and Welfare (AIHW) National Hospital Morbidity Database provides collated national data that enables trends in hospital admissions over time to be monitored. Such total-population data has the advantage of being unbiased and includes those disadvantaged groups often missed in traditional studies [9]. The aim of this study was to use total-population data to analyse trends in hospital separations with a primary diagnosis of an orofacial cleft over a 10-year period in Australia. Average length of stay (LoS) and admission rates by different age groups, gender and year were also investigated.

## Methods

This study used total-population hospital data for Australia with state data merged by the AIHW to produce national total-population data. Data on admissions between 1^st^ of July 2000 and 30^th^ of June 2010 (i.e. across financial years) of people of all ages to public and private hospitals across all Australian states and territories with a principal diagnosis of cleft lip and/or palate were accessed online from the AIHW National Hospital Morbidity Database [7]. This database contains information collected from hospitals on every episode of care from admission to discharge, transfer or death (defined as a separation). It should be noted that patients who were admitted to hospital more than once that had more than one separation (or record) in the database and individuals could not be identified, only number of separations. The information retrieved in this study included the total number of separations per year with a principal diagnosis of cleft lip/palate, the LoS, gender and age. The principle diagnosis is defined as the diagnosis established to be chiefly responsible for the admitted patient’s episode of care. The data are typically recorded in International Statistical Classification of Diseases and Related Health Problems, 10^th^ Revision, Australian Modification (ICD-10-AM) diagnosis codes. The three ICD-10-AM codes of interest in this study were Q35 – cleft palate (CP), Q36 - cleft lip (CL) and Q37 - concurrent cleft lip and palate (CLP). Some further levels of coding (and hence details of diagnosis) were available but beyond the scope of this study.

Estimated resident population (ERP) counts of all socio-demographic stratifications and age groups from the financial years of 2000 to 2010 are available from the 2012 Australian Demographic Statistics Report released by the Australian Bureau of Statistics [10]. The ABS frequently re-estimates population sub-groups using census data collected in more recent years by taking births, deaths and rates of overseas migration into account. Admission rates were calculated by firstly dividing the number of admissions by the ERP over the given time period, then multiplying by 10,000 to extrapolate the number of hospital admissions per 10,000 people per year.

Simple descriptive statistics were used to describe the data. Trends over time of the different cleft types, stratified by gender, were investigated by negative binomial regression using Stata v13.1 (StataCorp. 2013. Stata Statistical Software Release 13. StataCorp LP College station, TX, USA). This project used publicly available raw data, not available for person as unit of analysis, and therefore no ethics approval was required, an exemption from review was obtained from the Human Research Ethics Committee of the University of Western Australia (RA/4/1/7865).

## Results

Over the period of the study 11,618 separations were recorded across Australia with a principal diagnosis of an orofacial cleft with (ICD-10-AM code of Q35-Q37). The most common diagnosis for these separations was CLP (Q37) with 4,913 separations (0.25 per 10,000 people per year) followed by CP (Q35) with 4,454 separations (0.22 per 10,000 people per year). The least common was CL (Q36) with 2,251 separations (0.11 per 10,000 people per year) (Table 1). There was a gradual decrease in the rates of admission with age across all cleft types particularly beyond the 20**–**24 age category i.e. 25 years of age and older. Over half (54.9 %) of all the admissions occurred in under one year olds, while 5.4 % (*n =* 632) were over the age of 25 years. The admission rate with a diagnosis of CLP was consistently greater than both CL and CP across all ages groups up to 25 years with the exception of the 1–4 year old age group, where the highest admission rate was for infants with a diagnosis of CP. Admission rates from 25 years and older were low with no apparent differences across cleft type.

**Table 1:** Hospital admission rates by age group for orofacial clefts (ICD-10-AM: Q35-37) in Australia inclusive of 2000-2010

Age	Cleft Palate		Cleft Lip		Cleft Lip and Palate	
	*N*	*Rate*	*N*	*Rate*	*N*	*Rate*
<1	2507	9.32	1271	4.73	2606	9.69
1–4	931	0.87	210	0.20	387	0.36
5–9	395	0.29	182	0.14	370	0.27
10–14	213	0.15	132	0.09	556	0.40
15–19	143	0.10	217	0.15	512	0.36
20–24	84	0.06	105	0.07	255	0.17
25–29	30	0.02	35	0.02	50	0.03
30–34	25	0.02	23	0.02	46	0.03
35–39	28	0.02	21	0.01	34	0.02
40–44	26	0.02	21	0.01	28	0.02
45–49	20	0.01	8	0.01	25	0.01
50–54	15	0.01	13	0.01	15	0.01
55–59	8	0.01	5	0.004	14	0.01
60–64	11	0.01	4	0.004	7	0.01
65–69	10	0.01	1	0.001	6	0.01
70–74	7	0.01	3	0.005	1	0.002
75–79	1	0.002	0	0	1	0.002
Total	4454	0.22	2251	0.11	4913	0.25

Rate = Admission rates per 10,000 people per year based on estimated resident population (ERP) (July 2000 to June 2010)

Between June 2000 and 2010 more males (*n =* 6,510) than females (*n =* 5,108) were admitted to hospitals for procedures related to an orofacial cleft (Table 2). Amongst the male admissions, CLP was the most common diagnosis accounting for 3,031 or 46.6 % of the total 6,510 male separations whereas CP was the most common (45.2 %) cleft diagnostic code associated with admissions in females. Over this 10-year period, admission rates fluctuated slightly but there was little overall change in any of the diagnostic groups. Statistically significant downward trends over time were identified for admissions of female for CP (*p <* 0.0270), female for CL (*p <* 0.0081) and male for CLP (*p <* 0.0022).

**Table 2:** Hospital admission rates by financial year for males and females aged 0 ≤ 79 with orofacial clefts (ICD-10-AM: Q35-37) in Australia

Year	Cleft Palate			Cleft Lip			Cleft Lip and Palate			Total
	Male	Female^a^	Combined	Male	Female^a^	Combined	Male^a^	Female	Combined	Rate *(N)*
	*Rate (N) Rate (N)*	*Rate (N)*	Rate *(N)*	*Rate (N)*	*Rate (N)*	Rate *(N)*	*Rate (N)*	*Rate (N)*	Rate *(N)*	
	*Rate (N) Rate (N)*	*Rate (N)*	Rate *(N)*	*Rate (N)*	*Rate (N)*	Rate *(N)*	*Rate (N)*	*Rate (N)*	Rate *(N)*	
2000–01	0.25 *(206)*	0.25 *(232)*	0.23 *(438)*	0.15 *(138)*	0.10 *(92)*	0.12 *(230)*	0.3 (286)	0.17 *(162)*	0.24 *(448)*	0.59 *(1116)*
2001–02	0.23 *(216)*	0.22 *(209)*	0.22 *(425)*	0.14 *(138)*	0.12 *(112)*	0.13 *(250)*	0.31 (296)	0.19 *(177)*	0.25 *(473)*	0.60 *(1148)*
2002–03	0.23 *(220)*	0.24 *(233)*	0.24 *(453)*	0.12 *(119)*	0.10 *(97)*	0.11 *(216)*	0.30 (285)	0.19 (*178)*	0.24 *(463)*	0.59 *(1132)*
2003–04	0.22 *(215)*	0.27 *(262)*	0.25 *(477)*	0.16 *(158)*	0.10 *(95)*	0.13 *(253)*	0.32 (315)	0.20 *(195)*	0.26 *(510)*	0.64 *(1240)*
2004–05	0.20 *(201)*	0.26 *(256)*	0.23 *(457)*	0.13 *(126)*	0.10 *(94)*	0.11 *(220)*	0.33 (330)	0.21 *(205)*	0.27 *(535)*	0.62 *(1212)*
2005–06	0.20 *(196)*	0.24 *(236)*	0.22 *(432)*	0.11 *(110)*	0.08 *(79)*	0.09 *(189)*	0.27 (270)	0.17 *(165)*	0.22 *(435)*	0.53 *(1056)*
2006–07	0.19 *(194)*	0.21 *(214)*	0.20 *(408)*	0.15 (*150)*	0.08 *(84)*	0.12 *(234)*	0.30 (304)	0.19 *(196)*	0.25 *(500)*	0.56 *(1142)*
2007–08	0.19 *(197)*	0.21 *(216)*	0.20 *(413)*	0.10 *(109)*	0.07 *(77)*	0.09 *(186)*	0.37 (380)	0.19 *(200)*	0.28 *(580)*	0.57 *(1179)*
2008–09	0.23 *(241)*	0.21 *(226)*	0.22 *(467)*	0.14 *(145)*	0.09 *(90)*	0.11 *(235)*	0.30 (316)	0.20 *(209)*	0.25 *(525)*	0.58 *(1227)*
2009–10	0.24 *(260)*	0.21 *(224)*	0.23 *(484)*	0.13 *(140)*	0.09 *(98)*	0.11 *(238)*	0.23 (249)	0.18 *(195)*	0.21 *(444)*	0.54 *(1166)*
2000–10	*(2146)*	*(2308)*		*(1333)*	*(918)*		*(3031)*	*(1882)*		*(11618)*

Rate = Admission rates per 10,000 people per year based on estimated resident population (ERP)

^a^significant (*p <* 0.05) downward trend over time (negative binomial regression)

**Fig. 1:**
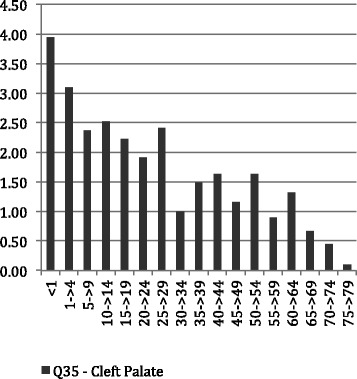
Average length of stay (days per admission) by age group for individuals with isolated cleft palate (ICD-10-AM: Q35) between 2000–2010

**Fig. 2:**
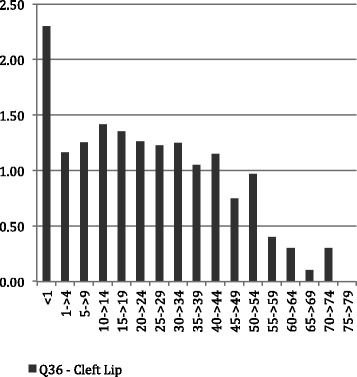
Average length of stay (days per admission) by age group for individuals with isolated cleft lip (ICD-10-AM: Q36) between 2000–2010

**Fig. 3:**
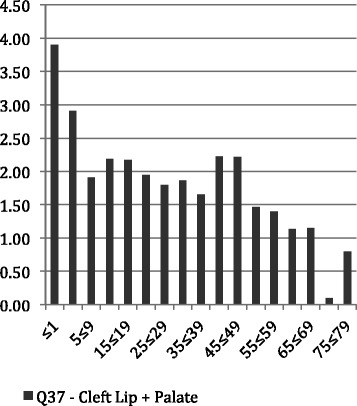
Average length of stay (days per admission) by age group for individuals with concurrent cleft lip and palate (ICD-10-AM: Q37) between 2000–2010

**Fig. 4:**
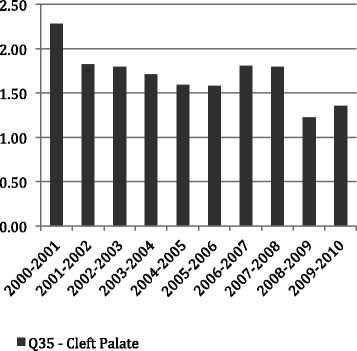
Average length of stay (days per admission) by year for individuals with isolated cleft palate (ICD-10-AM: Q35) between 2000–2010

**Fig. 5:**
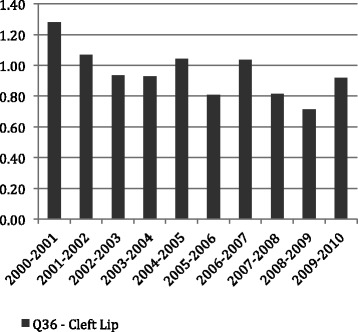
Average length of stay (days per admission) by year for individuals with isolated cleft lip (ICD-10-AM: Q36) between 2000–2010

**Fig. 6:**
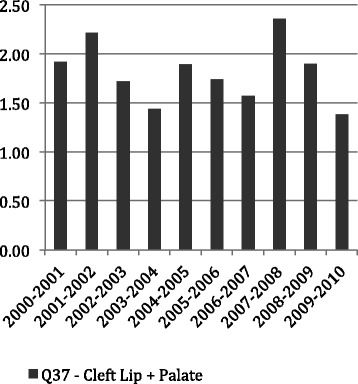
Average length of stay (days per admission) by year for individuals with concurrent cleft lip and palate (ICD-10-AM: Q37) between 2000–2010

Figures 1, 2 and 3 show the average LoS per admission to hospital by age throughout the period of the study for all admissions with principal diagnosis of an orofacial cleft. In general, for younger age groups at time of admission there was a longer LoS. Admissions for CLP and CP had a longer average episode of care, than those with CL. In particular, the 1–4 year old age group with CL spent less than half the time (1.16 days) in hospital compared with both the CLP (2.91 days) and CP (3.09 days). This difference was similar in the youngest children but, while maintained across the life-course, did reduce with age. Figures 4, 5 and 6 summarise the average length of stay, by each cleft type, from 2000 to 2010. While no clear trend was observed across the 10 year study period, there was a tendency for the LoS to reduce in both the CL and CP diagnostic groups.

## Discussion

This study provided an important opportunity to explore the trends in hospital admissions with a diagnosis of cleft of the lip/palate in Australia using total population data. A total of 11,618 episodes of care (or separations) were identified with a primary diagnostic code of orofacial clefting (ICD-10-codes Q 35, 36 and 37) over a 10 year period from July 2000 to June 2010. While it is likely that the majority of these separations represent cleft related procedures or surgeries, caution is required in any assumptions beyond the information available. It is possible, within this dataset, that a separation would have been recorded for an individual who had been admitted to hospital with a cleft primary diagnostic code and then discharged without having undergone any procedure (due to illness or a hospital initiated postponement). Primary closure of a cleft of the lip (with or without a cleft palate), is generally carried out within the first six months of life [11] and primary palate closure, with the exception of some infants with co-existing morbidities (such as Robin Sequence or other significant medical issues), is recommended by the age of 18 months [3]. Moving through early childhood in to adolescence, the higher admission rate for those with a combined CLP diagnosis compared to that of their peers with CP or CL may be reflective of the need for secondary alveolar bone grafting and later orthognathic procedures in this group [12, 13].

The identification of over 600 separations (over 25 years of age) admitted with a principal diagnosis of CLP over the 10 year time period of this study is worthy of further exploration. International standards suggest that the cleft care pathway is generally complete by late adolescence or early adulthood, with orthognathic surgery and secondary cosmetic revision to the lip and nose procedures being undertaken once growth has ceased [14, 15]. However these data suggest that there is a group of individuals who continue to require cleft related hospitalisation throughout their lives. It is likely that these hospitalisations are related to additional surgical procedures including treatment of persistent oronasal fistulae and nasal deformities as well as further cosmetic jaw surgery. Little is known about the longer term needs of older individuals living with a cleft of the lip and/or palate, however there is evidence that many affected adults are, or become, dissatisfied and seek further surgical, orthodontic and/or dental treatment to improve their appearance or function [16, 17]. However it is also possible that this older population may be at risk of developing certain co-morbidities in later life, such as mental ill health or cancer, which may necessitate hospitalisation [18, 19]. Internationally most cleft teams are based within paediatric healthcare settings and access to appropriately experienced clinicians to plan and treat this complex population of older individuals remains a challenge [17].

The rates of admission for CP (0.22/10,000 ERP) or CLP (0.25/10,000) were twice those of the CL group (0.11/10,000) across the entire age range. Despite the lack of Australia wide birth defects registry data, these admission rates are reflective of what is known about the epidemiology of orofacial clefting. Recent data from the Western Australia Registry for Developmental Anomalies [8] show that, over the period 1980 to 2009, the birth prevalence rates of babies in Western Australia born with CP were twice those of both CL and CLP (CP: 10.12/10,000: CL: 5.09/10,000 births, CLP: 6.99/10,000). The relatively high admission rate of CLP (0.25/10,000 ERP) which was actually slightly greater than for CP (0.22/10,000 ERP) may be explained by the fact that this group usually require many more surgical procedures than either isolated CP or CL. Over the 10 year period there was no overall change in rates of admission in any of the cleft groups. This lack of change is underpinned by a similar lack of change in the birth prevalence rates reported in either Western Australia or Victoria over recent years [8, 20].

There is often pressure on health services to reduce the length of time patients spend in hospital however there was no significant reduction in LoS for any of the cleft types over the 10 year period. Such reductions in LoS have been reported recently for children undergoing primary surgery for clefts of all types in the UK [11]. A study by Shmueli and Savage in 2014 compared the hospitalisation characteristics of private and public patients discharged from hospitals in New South Wales during 2004–2005. They identified that while private patients have one third less waiting days than public patients, the length of stay are unrelated to the insurance status [21], however it is not clear if this trend also relates to cleft admissions. How long a child remains in hospital may therefore be influenced by a variety of factors including the severity of cleft, additional congenital malformations, post-operative complications and clinician preference [22, 23]. These factors may explain the shorter (almost half) LoS for infants born with CL because this type of cleft is rarely associated with other co-morbidities or syndromes [24, 25] and the surgery is considered relatively straight forward [22]. It was, however, not surprising to find that infants under the age of 12 months, spent longer in hospital than older children.

## Limitations

The data collected from this study were total-population data. However as with all use of administrative data we are dependent on the quality and detail of the data supplied. While it is likely that the majority of these separations represent cleft related procedures or surgeries, it is possible within this dataset, that a separation would have been recorded for an individual who had been admitted to hospital with a cleft primary diagnostic code and then discharged without having undergone any procedure (due to illness or a hospital initiated postponement). Similarly an individual may be admitted to hospital for an emergency e.g. a fractured limb or other non-cleft related reason and still be assigned a primary cleft diagnostic code. Nevertheless it is likely that the majority of these separations are associated with cleft related procedures given the principal diagnosis codes. This assumption is supported by the high rates of admission for very young infants with the majority of all separations occurring in the under five year olds, and with the under 12 month olds representing over half the total admissions.

Although it is possible to analyse limited further details of these diagnosis codes, this study focused only the broad (typically used) categories of diagnosis and small numbers may not allow investigation at more detailed level. It is also not possible to classify differences in orofacial clefts as severity, specific forms or syndromic or non-syndromic from the data evaluated in this study and further investigation will be valuable as details in total-population data improve.

## Conclusions

Quantifying service utilisation has a role to play, not only in planning and commissioning of health services but in monitoring outcomes and improving care. A particular strength of this study is the population-level information on admissions to all hospitals in Australia for each of the three main cleft conditions; cleft lip, cleft lip and palate and cleft palate. These data have been analysed to better understand the patterns of the separations according to characteristics such as age, gender and length of stay. Analysis of such large scale datasets optimises the generalizability of the results and ensures that no one is excluded [9]. An additional strength is the ability to observe trends across the lifespan of this population. This study has identified a small but significant number of cleft related separations in the 25 year and older age groups. As the population of Australia ages, understanding the needs and expectations of this group is important not only for those individuals today but for future generations to come.

## Availability of data and materials

Raw data of separation statistics collected for this paper can be accessed online through the Australian Institute of Health and Welfare website. More specifically, the National Hospital Morbidity Database (http://www.aihw.gov.au/hospitals-data/principal-diagnosis-data-cubes/) has separation statistics from 1993-2014. The provided link has access to an SAS Web Report Studio which allows filtering of data that can be exported. Raw data of Estimated Resident Population for Australia can be accessed online through the Australian Bureau of Statistics website. This information can be exported through the Australian Demographic Statististics datacubes (http://www.abs.gov.au/ausstats/abs@.nsf/mf/3101.0/) for all states and territories.

## Abbreviations

AIHW: Australian Institute of Health and Welfare
CL: Cleft lip
CLP: Cleft lip and palate
CP: cleft palate
ERP: Estimated resident population
LoS: Length of stay
